# Chronic diarrhea as the presenting feature of primary systemic AL amyloidosis: serendipity or delayed diagnosis?

**DOI:** 10.1186/1471-230X-13-71

**Published:** 2013-04-24

**Authors:** Chen Wang, Yue Li, Yali Jin, Weixun Zhou, Yanlin Zhu, Fang Yao, Jiaming Qian

**Affiliations:** 1 Department of Medicine, Peking Union Medical College, Beijing, China; 2 Department of Gastroenterology, Peking Union Medical College Hospital, No.1 Shuaifuyuan, Beijing, 100730, China; 3 Department of Pathology, Peking Union Medical College Hospital, Beijing, China; 4 Department of Cardiology, Peking Union Medical College Hospital, Beijing, China

**Keywords:** Chronic diarrhea, Amyloidosis, Primary systemic AL amyloidosis

## Abstract

**Background:**

Chronic diarrhea in adults is a common symptom with a wide range of underlying etiologies. Although various strategies have been proposed for evaluation, there are still cases with undetermined origins even after extensive workup. Amyloidosis with gastrointestinal (GI) involvement is one of the causes that should be considered in adult patients with chronic diarrhea. We report a case of primary systemic amyloid light-chain (AL) amyloidosis, presenting initially as chronic diarrhea and weight loss.

**Case presentation:**

A 43-year-old man with chronic diarrhea and weight loss was referred to our hospital. Prior to his presentation, extensive evaluation including an exploratory laparotomy was carried out and did not yield any valuable findings. An echocardiography performed after repeated episodes of orthostatic hypotension revealed infiltrative cardiomyopathy. Moreover, biopsies of the terminal ileum revealed amyloid deposition confirmed by Congo Red staining. Finally, a diagnosis of systemic AL amyloidosis was made after hematological workup. Anti-plasma cell therapy did ameliorate his GI symptoms.

**Conclusion:**

Although amyloidosis with GI involvement is a rare cause of chronic diarrhea, it should be considered especially in patients with intestinal malabsorption and extra-GI manifestations, such as orthostatic hypotension. The delayed diagnosis in the present case highlights the importance of recognizing clinical “red flags” not seemingly related to one another, and underscores the need to get intestinal biopsies even with normal endoscopic appearance of the mucosa.

## Background

Chronic diarrhea is common with a prevalence of 3% to 5% in the general population [1]. The major causes of chronic diarrhea can be assessed by combining a patient’s history and characteristic of the stool (inflammatory, fatty or watery). The differential diagnosis covers a wide range of diseases from common encountered entities such as chronic infection, inflammatory bowel disease, laxative abuse, and irritable bowel syndrome to rare disorders. Guidelines recommend categorizing chronic diarrhea as osmotic, inflammatory, secretory or motile, which help focus the diagnosis and further tests. Despite careful evaluation of the differential diagnosis of chronic diarrhea, physicians can sometimes fail to recognize the cause. In this case, physicians should take into consideration causes less frequent or even rare diseases. In the present report, a case of primary AL amyloidosis with GI involvement presented as chronic diarrhea and weight loss. Although similar conditions have been reported scantly [2,3], none of them was initially diagnosed via random intestine biopsy, and with a detailed therapeutic follow-up.

## Case presentation

A 43-year-old man developed watery diarrhea that gradually worsened over two years as well as progressive weight loss of 35 kg.

He had an average stool frequency of up to 6 to 8 times a day, with no obvious blood or mucus and no abdominal pain or tenesmus. He had no fever, night sweats, nausea, vomiting, hematochezia, or melena. Migrating tingling of both lower extremities was noticed one and a half years earlier. During the past two years, he had undergone both esophagogastroduodenoscopy (EGD) and colonoscopy twice with normal appearance of the GI mucosa. Biopsy of the intestine was not performed. Small bowel follow-through as well as abdominal computed tomography (CT) was unremarkable. In addition, an exploratory laparotomy was performed 1 year ago with no diagnostic findings. It is unknown whether in the previous period postural hypotension had been observed or not. And the presence of cardiomyopathy, sensorimotor peripheral neuropathy and micturition disorders were also not highlighted in previous medical referrals.

The past medical history was remarkable for a diagnosed tuberculous pericarditis twenty years ago. Half a year later, no obvious abnormality was detected by echocardiography after treatment with standard anti-tuberculosis regimen. No hypertrophic cardiomyopathy was noticed.

On physical examination, his blood pressure was 94/63 mmHg and pulse rate was 80 beats per minute. A mild systolic murmur, grade 3/6, was heard at the aortic valve area. The abdominal exam was normal and no lymphadenopathies were found.

Laboratory studies revealed anemia (hemoglobin 95 g/L, reference value 120–160 g/L) with normal mean corpuscular volume (97.5 fL) of red blood cell, positive Sudan III staining of the stool, mildly increased levels of serum creatinine (120 μM, reference value 59–104 μM). Low density lipoprotein was low (1.90 mM, reference value 2.07-3.63 mM). Iron studies revealed a low serum iron level (59.2 μg/dl, reference value 65–175 μg/dl), a slightly low total iron-binding capacity (273 μg/dl, reference value 280–430 μg/dl) and an elevated ferritin level (669 ng/ml, reference value 24–336 ng/ml). A D-xylose test revealed a total excretion of 0.4 g in the five-hour collection of urine (reference value more than 1.2 g). Serum electrolytes, total protein, albumin, bilirubin, alkaline phosphatase, alanine aminotransferase, γ-glutamyl transferase, glucose, amylase, and lipase were all within normal limits. Microbiologic and parasitologic analyses of the stools were negative. Antinuclear, anti-gliadin, anti-reticulin and anti-endomysial antibodies were all negative. The patient was appropriately evaluated for the most common endocrinologic causes of diarrhea. Diabetes mellitus, hyperthyroidism and adrenal insufficiency were excluded with the available medical history and laboratory tests. For a rare, but important class of chronic secretory diarrheal illness caused by peptide-secreting tumors (e.g. carcinoid tumors, VIPoma, gastrinoma), there was no remarkable suggestions after extensive biochemical tests and imaging studies (CT scan and somatostatin receptor scintigraphy).

The available data and the clinical picture first led us to suspect an osmotic diarrhea associated with malabsorption. However, even after a careful consideration of various causes of diarrhea associated with malabsorption, it was not possible to identify a cause that could confirm the suspected diagnosis. Among the most common disorders are lactose intolerance, giardiasis, celiac disease and bacterial overgrowth of the small intestines [4]. Rarely, Whipple’s disease, microscopic colitis, eosinophilic gastritis and lymphoma also manifest as chronic diarrhea [5]. In these cases several random biopsies of the intestinal mucosa during EGD and ileocolonoscopy are often decisive in highlighting the pathological process of malabsorption.

**Figure 1:**
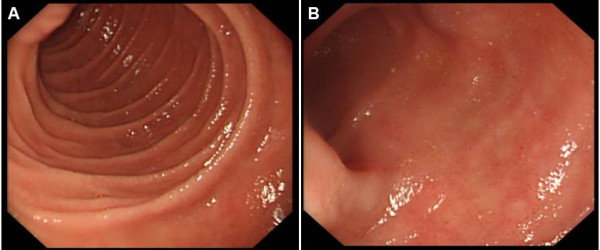
**Gastrointestinal endoscopic findings of the patient.** Normal appearance of the second part of duodenum (**A**) and the terminal ileum (**B**).

**Figure 2:**
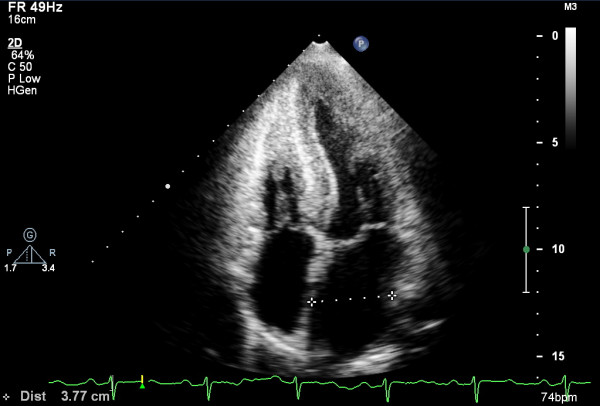
**Echocardiographic findings of the patient.** Apparent left ventricular hypertrophy and “snowstorm” appearance of the myocardium are suggestive of an infiltrative cardiomyopathy.

**Figure 3:**
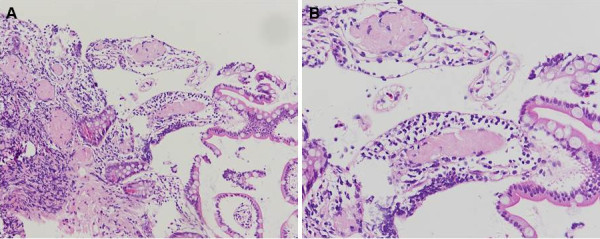
**Microscopic findings of the terminal ileum biopsy.** Hematoxylin and eosin staining shows extracellular deposits of eosinophilic amorphous material consistent with amyloid. Original magnification, ×100 (**A**) and ×200 (**B**).

EGD and ileocolonoscopy revealed normal appearance of the GI mucosa (Figure 1). Before scheduled endoscopy examination, the patient complained of several episodes of dizziness when he passed from the supine to the upright position. Orthostatic hypotension was confirmed and an echocardiography was ordered to exclude heart disease. The echocardiographic findings showed a hypertrophic cardiomyopathy with a characteristic ground-glass appearance and thickening of the interventricular septum (Figure 2). The random biopsies from the terminal ileum revealed in the intestinal mucosa an eosinophilic amorphous material (Figure 3). The deposits resulted positive for Congo red staining, even pretreated with potassium permanganate before Congo red staining. Potassium permanganate treatment is a simple way to evaluate the chemical type of amyloid protein. Such a pretreatment does not affect staining affinity for Congo red in AL amyloid but eliminates this affinity in AA amyloid, although not in all cases [6]. The same biopsy examined under polarized light revealed apple-green birefringence, typical of amyloid. Therefore we confirmed the diagnosis of systemic amyloidosis with GI and cardiac involvement.

Primary or reactive amyloidosis was further differentiated. Protein electrophoresis showed a narrow band formed by paraprotein (M band). Immunofixation demonstrated it as IgG-lambda type in both serum and urine specimens. Further laboratory studies revealed decreased serum immunoglobulin levels (IgA 0.35 g/L, reference value 0.7-3.8 g/L; IgM 0.26 g/L, reference value 0.6-2.5 g/L), elevated free lambda light-chain levels, and an abnormal ratio of free kappa to free lambda light chains. Myelogram revealed a marrow plasmacytosis of 11.5%. X-rays did not show any skeletal osteolytic lesions. The final diagnosis was systemic AL amyloidosis IgG lambda-type BJ positive lambda.

Before the final diagnosis was reached, the control of the diarrhea (a stool frequency of 1 to 2/day) was attained with diosmectite and loperamide. Then, after the diagnosis was made, a chemotherapy regimen (bortezomib, dexamethasone and thalidomide) was initiated and continued for four courses, achieving a good clinical response. The stool frequency was 1 to 2/day after discontinuation of anti-diarrhea medications. Serum creatinine level was around 80 μM. At the last follow up, the patient had gained 6 kg in weight.

## Conclusions

Amyloidosis is a generic term that refers to a heterogeneous group of diseases, characterized by the deposition of misfolded fibrils in various tissues. In each type of amyloidosis, a different protein produced by the body acquires the property to accumulate in different organs and tissues in the form of fibrils. Systemic AL amyloidosis is the most common type, associated with plasma cell dyscrasia, which produces immunoglobulin light chain that is amyloidogenic. The secondary amyloidosis is due to amyloid formed from serum amyloid A (AA), an acute-phase protein produced in response to inflammation. For familial amyloidosis, the most frequent type seen on tertiary referral centers is caused by mutant transthyretin (TTR) deposition. The clinical manifestations depend on the number and nature of organs affected, in which GI involvement is quite rare. GI amyloidosis is more common in reactive amyloidosis, but was also reported in primary amyloidosis, with prevalence about 1% in a retrospective cohort [7]. Primary amyloidosis usually presents with constipation, mechanical obstruction, while reactive amyloidosis presents with diarrhea and malabsorption [8]. However, for this patient, GI involvement did explain his chronic diarrhea and malabsorption. Clinical symptoms vary in accordance with the layer of bowel wall involved. Mucosa predominant disease is manifested as malabsorption, whereas muscularis predominant disease presents as obstruction. Autonomic neuropathy may also affect gut function, which was distinguished in GI symptoms caused by mutant transthyretin amyloidosis [9]. In our patient, the malabsorption was considered as a comprehensive result of his specific pathological changes. In addition, the orthostatic hypotension and moderate renal insufficiency suggested cardiac and renal involvement. The migratory tingling in the history may be also due to peripheral neuropathy-induced by amyloid deposition.

After the diagnosis was established treatment was directed to the hematological cause, namely plasma cell dyscrasia. GI symptoms, especially diarrhea, were greatly improved after four courses of chemotherapy, which should be mainly attributed to the anti-plasma cell therapy with decreased production of amyloid proteins [10]. Also noteworthy is the need to maintain special precautions in the use of thalidomide and bortezomib for their common adverse effects reducing the intestinal motility [11]. The renal insufficiency of this patient also remitted, since bortezomib has become a standard regimen therapy for these patients [12].

In summary, the diagnostic proceeding of this patient emphasizes the need of a stepwise integration of results from detailed medical history, physical examination, laboratory and imaging studies, focusing generally on the malabsorption syndrome. Even in this way, the diagnosis of amyloidosis is still difficult to confirm. The delay from onset of symptoms to diagnosis was from 7 to 24 months in a retrospective study and could be due either to the patient’s or to the physician’s delay [13]. The reason for the physician’s delay is only partly due to the rarity of the disease. In fact, the onset of clinical signs or symptoms at different times considered to be non-specific will become decisive factors for the orientation towards a correct diagnosis, if the doctor could relate them to each other. Panels of differential diagnoses are certainly very useful. It should be stressed that some symptoms (if reported or recognized), seemingly unrelated to each other, are actually early and specific red flags of the amyloid process. In this patient, the differential diagnosis was transformed from a broad question into a narrow one after the orthostatic hypotension was noticed. However, other characteristic clinical signs of a possible amyloidosis could have been recognized previously in addition to malabsorption and diarrhea (gastrointestinal illness). These include migrating tingling of lower extremities (neuropathy disease), hypertrophic cardiomyopathy (heart disease), renal failure (kidney disease), anemia and monoclonal gammopathy (hematologic disease). Each of these aspects can cause a patient to consult doctors of different specialties, making it difficult to achieve an accurate early diagnosis. Increasing medical alertness of the natural history of the systemic amyloidosis could reduce the time to achieve the diagnosis and therapeutic decisions, also improving the prognosis. Finally, it could allow physicians to reach the correct diagnosis earlier and achieve a (serendipitous) positive biopsy result obtained from the random intestinal biopsy even with normal endoscopic appearance.

Moreover, it is useful to emphasize that even without histological evidence in the first diagnostic approach, if the clinician has a solid suspect of amyloidosis, before abandoning this hypothesis, it is necessary to verify data with “perseverance”, in order to control the correct criteria and results with ambiguous diagnostic evidence.

### Consents

Written informed consent was obtained from the patient for publication of this case report. A copy of the written consent is available for review by the Editor-in-Chief of this journal.

## Abbreviations

EGD: Esophagogastroduodenoscopy; GI: Gastrointestinal; AL: Amyloid light-chain; CT: Computed tomography; AA: Amyloid A.

## Competing interests

The authors declare that they have no competing interests.

## Authors’ contributions

CW collected the references and wrote the manuscript. YJ collected the clinical data. WZ analyzed the pathological results. YZ performed echocardiographic examination. YL initiated the paper, helped to incorporate the clinical data into the manuscript and mentored the writing and completed the revisions of the manuscript. FY and JQ mentored the study. All authors report no conflicts of interest and approved the final manuscript.

## Pre-publication history

The pre-publication history for this paper can be accessed here:

http://www.biomedcentral.com/1471-230X/13/71/prepub
